# Low genetic differentiation yet high phenotypic variation in the invasive populations of *Spartina alterniflora* in Guangxi, China

**DOI:** 10.1371/journal.pone.0222646

**Published:** 2019-09-17

**Authors:** Fei-Fei Li, Lu Gong, Jun-Sheng Li, Xiao-Yan Liu, Cai-Yun Zhao

**Affiliations:** State Key Laboratory of Environmental Criteria and Risk Assessment, Chinese Research Academy of Environmental Sciences, Beijing, P.R. China; Brigham Young University, UNITED STATES

## Abstract

Genetic variation and population structure may reflect important information for invasion success of exotic plant species and thus help improve management of invasive plants. *Spartina alterniflora* is an invasive plant that is a major threat to the economy and environment of the coastal regions in China. We analyzed the genetic structure and diversity of six populations of *S*. *alterniflora* differing in invasion histories in Guangxi, China. A total of 176 individuals from the six populations produced 348 AFLP fragments. The average heterozygosity was significantly lower than in the native population. And genetic bottlenecks were also detected in most populations. Standardized *F*_*ST*_ statistics (*Φpt* = 0.015) and AMOVA results indicated weak genetic differentiation. Genetic admixture and obviously isolation by distance indicated populations in Guangxi come from a pre-admixed population by a single introduction. High phenotypic variations of *S*. *alterniflora* in Guangxi influenced by soil salinity and temperature might be an important reason for the successful invasion.

## Introduction

Under the development of global trade and tourism, invasive alien species became one of the main direct drivers of biodiversity loss across the globe. The founding events cannot prevent some introduced species from successful colonization and rapidly spreading. They can become highly invasive despite having gone through genetic bottlenecks and low genetic diversity [1, 2]. Such an invader’s success may be attributed to multiple introductions [3], asexual reproduction [2], the presence of pre-adapted genotypes [4, 5], and high levels of morphological plasticity [6]. Therefore, multiple associated factors should be comprehensively considered when studying the invasion mechanisms of invasive alien species.

*Spartina alterniflora* Loisel., native to the Atlantic and Gulf Coast estuaries of North America, is listed as one of the most invasive plants in China [7]. This perennial grass is commonly located in lower intertidal salt marshes and has the capacity to reduce shoreline scouring and trap sediments [8, 9]. It has been introduced deliberately or accidentally to the coasts of England, France, and other countries or regions [10–13]. It has become a problematic species as it can out-compete native plants, cause estuary channel siltation, and change ecosystem structure [10, 14–16].

*S*. *alterniflora* was first introduced into Fujian of China for land-building and tideland restoration projects from three locations in North America (North Carolina, Georgia, and Florida) in 1979 [17]. Recently, its geographical range in China has expanded to Guangxi Province (*N* 20°54′-26°24′, *E* 104°28′-112°04′) in the south and to Liaoning Province (*N* 38°43'-43°26', *E* 118°53′-125°46′) in the north. It is now widely distributed over the Pacific coast of China, occupying 112,000 ha in 2000 [14, 18, 19]. The spread of *S*. *alterniflora* in China was much faster than that of the admixed populations in Willapa Bay [20, 21] and *S*. *alterniflora* × *S*. *foliosa* hybrids in San Francisco Bay [13, 21, 22]. However, studies revealed that the genetic diversity of *S*. *alterniflora* in China was lower than that in native populations at both the species and the population level [13, 23, 24]. Deng et al. [23] proposed that high genetic differentiation within populations and strong adaptability might promote the widespread of *S*. *alterniflora*. Xia et al. suspected coexistence of various intraspecific hybrids and mixtures from the three ecotypes sampled from their native ranges was considered as the main reason for the widespread [24]. Based on the broader comparisons of genetic structure between Chinese and native populations, Bernik et al. [13] found significant genetic differentiation between source populations but not among Chinese populations, and post-introduction admixture in China sites might result from mixed nursery stock or repeated introductions. Moreover, they didn’t find recent genetic bottlenecks in Shanghai and Zhejiang sites [13]. Such a genetic admixture of divergent intraspecific lineages and hybridization between species or subspecies in the invaded range may reduce negative effects of genetic bottlenecks, and thus may adapt well to local conditions by increasing heterozygote frequency and producing novel genotypes [25–27]. Some researchers point out phenotypic plasticity in response to different environmental conditions was also important for plant species successful invasion [28–30]. Recently, Zhao et al. [31] have shown there were significant differences in phenotypic traits among *S*. *alterniflora* populations in China, and the temperature was the main influencing factor. Through a common-garden experiment, Liu et al. [32] found most differences in phenotypic traits were disappeared in the common garden indicated that phenotypic plasticity contributions to various plant traits of *S*. *alterniflora* in China rather than genetic differentiation. Although previous studies provided important information for invasion success of *S*. *alterniflora* in China through genetic and phenotypic data, it is still unknown how genetic diversity of *S*. *alterniflora* changes during its invasion process, and the relationships between genetic differentiation, environmental changes and phenotypes differences of *S*. *alterniflora* in China have not been evaluated.

In Guangxi, *Spartina alterniflora* was first introduced to the beaches of Shanjiao Village, Beihai City in 1980 [13]. After successful colonization in Shanjiao Village, it spreads quickly along the Dandou Sea coast. Due to the afforestation project in 1994, *S*. *alterniflora* was also introduced to the outer beach of Beijie Village from the Dandou Sea coast [33]. Over 30 years, this species expanded its distribution area from 1 to 389.2 ha in Guangxi [33]. In 2008, a new patch of *S*. *alterniflora* was found in Qingshantou Village, Beihai City [33], and another new patch was found in Dongwei Village, Beihai City in 2013 [34]. In addition, we found a third new patch in Fangchenggang City in 2017. It reflected *S*. *alterniflora* had constantly been invading new areas in Guangxi during the past 25 years. These differently-aged populations are applicable to study genetic diversity changes during the invasion process.

In this study, we chose six populations of *S*. *alterniflora* differing in invasion history in Guangxi Province, China. Combined with phenotypic and environmental data, we addressed three main questions: (1) How invasion history affects the genetic diversity of *S*. *alterniflora* populations in Guangxi? (2) Did these populations undergo genetic bottlenecks? (3) Whether there were links between genetic differentiation, phenotypes, and environments. We also expect to provide evidence to explain the rapid spread of *S*. *alterniflora* in Guangxi and suggestions for invasive species management, especially for newly-established populations.

## Material and methods

### Study locations and sample collection

**Fig 1:**
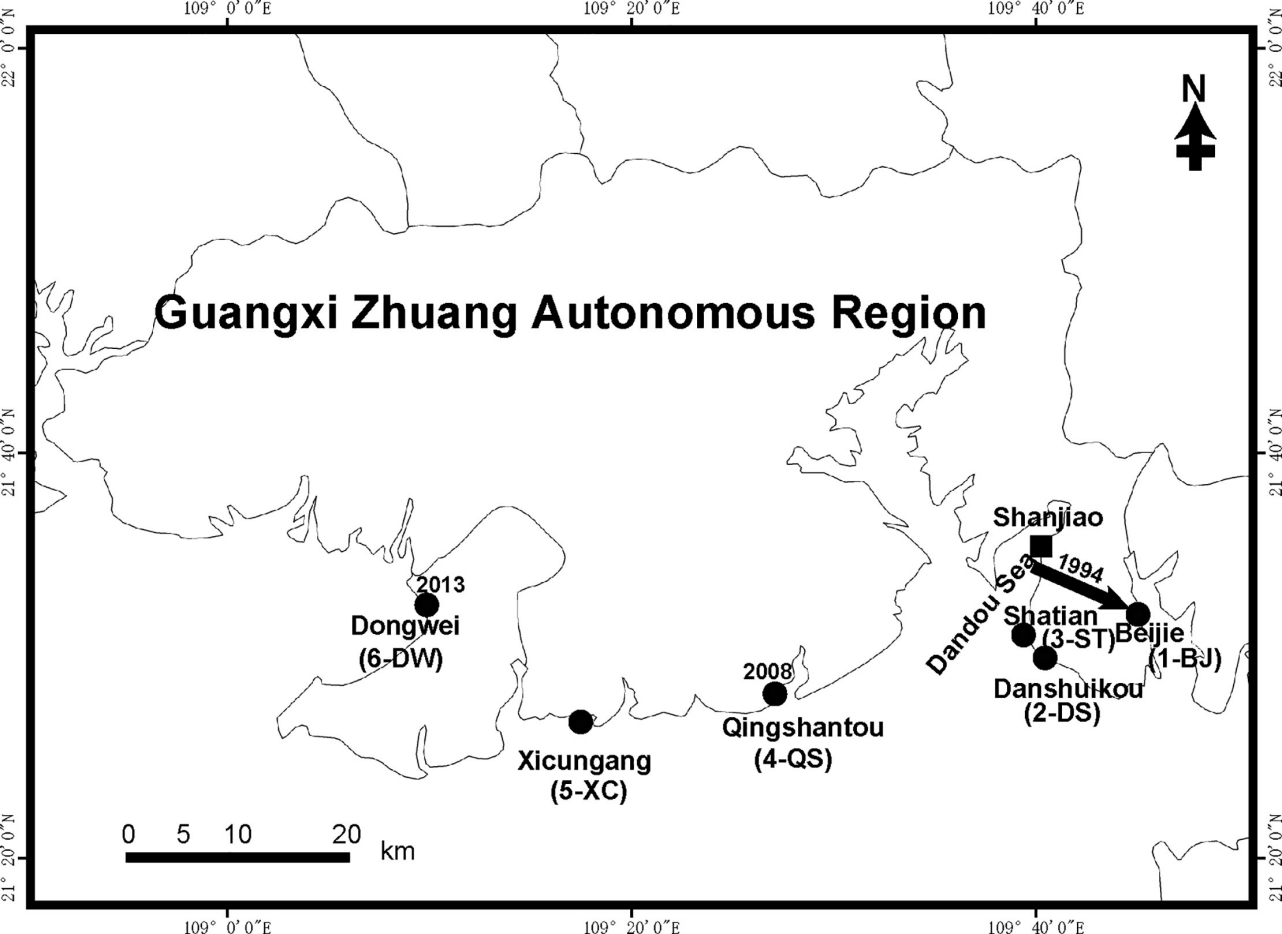
Introduction history and population location of *Spartina alterniflora* in Guangxi, China. This species was first introduced into Shanjiao Village (which belongs to the Dandou Sea coast) of Guangxi (GX) from Fujian (FJ) in 1980 and into Beijie (1-BJ) in 1994 from the Dandou Sea coast. The population in Qingshantou (4-QS) was first detected in 2008, and that in Dongwei (6-DW) was first detected in 2013.

We selected six populations of *S*. *alterniflora* along the coastal line of Guangxi, which differed in their invasion history. The populations were labeled using the abbreviated names of villages or harbors (1-BJ, 2-DS, 3-ST, 4-QS, 5-XC, and 6-DW). *S*. *alterniflora* in Beijie Village (1-BJ) was introduced from the Dandou Sea coast in 1994, and it has existed for more than 20 years. The populations at Danshuikou (2-DS) and Shatian Village (3-ST) were located near the first introduction site in the Dandou Sea coast. Population 4-QS was located on the coast of Qingshantou Village, on the west side of the Dandou Sea area. Population 4-QS was first detected in 2008 [33]. Population 5-XC was located at Xicungang harbor, to the west of 4-QS. Although we do not know the exact age of each population, *S*. *alterniflora* colonized in Qingshantou Village (4-QS), and Xicungang harbor (5-XC) were almost 14 years later than 1-BJ, 2-DS, and 3-ST, based on previous survey data and records. Population 6-DW was a newly-established population with a very small patch area, first observed in 2013. Population 6-DW was the newest population in our study. A total of 176 individuals were sampled from these populations in October 2013. The distance between any two samples was more than 10 m. The information on the number of individuals, location, and occupied area are listed in Table 1 and Fig 1.

**Table 1:** Location information, number of sampled individuals, genetic diversity, and recent bottlenecks of the six populations of *Spartina alterniflora* in Guangxi, China.

Pop.	Location	Latitude (*N*)	Longitude (*E*)	No.	*PPL*	*Na*	*Ne*	*H*	*I*	*P*_*L*_	*IAM*	
											*He/Hd*	*P*-value
1-BJ	Beijie Village	21.53°	109.76°	30	86.56	1.731	1.160	0.127	0.232	1.000	**198/124**	**<0.01**
2-DS	Danshuikou	21.50°	109.68°	30	84.14	1.683	1.140	0.114	0.212	2.000	**172/141**	**<0.01**
3-ST	Shatian Village	21.51°	109.66°	30	84.95	1.699	1.137	0.112	0.211	0.000	**173/143**	**<0.01**
4-QS	Qingshantou	21.47°	109.46°	30	84.95	1.699	1.136	0.109	0.205	0.000	**150/166**	**0.030**
5-XC	Xicun Harbor	21.43°	109.29°	30	83.33	1.667	1.123	0.101	0.192	4.000	141/169	0.120
6-DW	Dongwei Village	21.54°	109.17°	26	84.68	1.694	1.143	0.116	0.215	2.000	**152/163**	**0.010**
Mean	-	-	-	-	84.77	1.695	1.140	0.113	0.211	-	**-**	**-**
All	-	-	-	-	93.55	1.936	1.138	0.115	0.221	-	**261/87**	**<0.01**

No.: number of individuals; *PPL*: the percentage of polymorphic loci; *Na*: number of different alleles, *Ne*: number of effective alleles; *H*: average heterozygosity; *I*: Shannon’s information index; *P*_*L*_: number of private loci; *He/Hd*: ratio of the number of loci with a heterozygosity excess to the number of loci with a heterozygosity deficiency. *P*-values are determined by a sign test under the infinite allele model (IAM). Bottlenecks with significant results (*P*-value < 0.05) are highlighted in bold.

### DNA extraction and AFLP reactions

Genomic DNA was extracted from the leaves using the EasyPure Plant Genomic DNA Kit (Beijing TransGen Biotech Co., Ltd., Beijing, China) according to the manufacturer’s instructions. One leaf sample of approximately 20 mg was ground to a fine powder in liquid nitrogen, and 50 μL of elution buffer was added to dissolve the isolated DNA. The extracted DNA was quantified using a SMA4000 UV-Vis Spectrophotometer (Merinton, Beijing). The DNA integrity was determined using agarose gel electrophoresis (1% agarose; 1x TBE; 0.03 mg/ml Ethidium Bromide (EtBr)).

We made some modifications to the AFLP procedures [35]. For the digestion, 500 ng of genomic DNA was incubated at 37°C for 2 h in a 40-μL reaction, containing 4 μL of CutSmart Buffer, 10 U of *EcoR* I-HF and 5 U of *Mse* I (NEB, Beijing). The enzymes were inactivated at 65°C for 20 min. For the ligation, 10 μL of a ligation mix comprising 5.5 μL of digested DNA, 2 μL of ligase buffer, 1 μL of *EcoR* I-adapter (500 pM), 1 μL of *Mse* I-adapter (1000 pM), and 0.5 μL (100 U) of T4 DNA Ligase (TransGen Biotech, Beijing) were added to the samples and incubated at room temperature (25°C) for 1 h. The pre-selective polymerase chain reaction (PCR) was performed using primer pairs with a single selective nucleotide extension (*EcoR* I-A and *Mse* I-C). The reaction mix (total volume of 25 μL) comprised 2.5 μL of template DNA from the ligation step, 0.5 μL of primer (*EcoR* I/*Mse* I), and 12.5 μL of EasyTaq mix (TransGen Biotech, Beijing). The reaction included an initial incubation at 94°C for 2 min, followed by 20 cycles at 94°C for 20 s, 56°C for 30 s, and 72°C for 2 min, with a final extension at 72°C for 10 min. The PCR products of the pre-amplification reaction were used as the templates for selective amplification using two pairs of AFLP primer combinations with three selected nucleotides (*EcoR* I-ACG and *Mse* I-CTA, *EcoR* I-ACA and *Mse* I-CTA). The selective primers were labeled at the 5' ends using the fluorescent dye 6-FAM for the visualization of the fragments on the analyzer. The 25-μL selective amplification mix contained 1 μL of pre-amplification products, 12.5 μL of EasyTaq mix, and 0.5 μL (10 μM) of primers (3 selective primers, respectively). The reaction was conducted for 2 min at 94°C, followed by 10 cycles of 30 s at 94°C, 30 s at 65°C and 1 min at 72°C. The annealing temperature was reduced by 1°C per cycle. Then 20 cycles consisting of 30 s at 94°C, 30 s at 56°C, and 10 min at 72°C were performed. All amplification reactions were performed using a professional Standard 96-gradient Thermocycler (Biometra, Germany). The amplified fragments were separated, and the raw data were collected using capillary electrophoresis on an ABI3730XL Genetic Analyser (Applied BioSystems, USA) at the TsingKe Biotech Company (Beijing).

### Genetic diversity and genetic structure analysis

The raw data were processed using the fragment analysis software GeneMarker V2.2.0. The chromatograms of the fragment peaks were scored as present (1) or absent (0). A binary qualitative data matrix was constructed. GENALEX 6.503 [36] were used to estimate the number of AFLP fragments, the percentage of polymorphic loci (*PPL*), the number of different alleles (*Na*), number of effective alleles (*Ne*), average heterozygosity (*H*), Shannon’s information index (*I*), number of private loci (*P*), pairwise genetic differentiation (*Φpt*), gene flow (*Nm =* [(1/*Φpt*)-1]/2), and *Nei’s* genetic distance among populations. The analysis of molecular variance (AMOVA) was performed to estimate the allocation of genetic variation at three levels: among regions, among and within populations. The populations were partitioned into two regions: (1-BJ, 2-DS, 3-ST) and (4-QS, 5-XC, 6-DW) with the Dandou Sea as a partial barrier to gene flow. The dendrogram was constructed by the neighbor-joining method using MEGA-X [37], showing the genetic relationship among populations. We used a two-way analysis of variance (ANOVA) in the SPSS software to compare the average heterozygosity (*H*) between Chinese and native populations [9]. The difference of genetic diversity among populations in Guangxi was compared either.

Population structure was assessed using the Bayesian model-based clustering analysis with STRUCTURE 2.3 [38]. The initial range of potential genotype clusters (*K*) was specified from 1 to 6 with 10 independent runs under the admixed model at 100 000 MCMC iterations and a 10 000 burn-in period. The most probable number of clusters (*K*) was selected by calculating an adhoc statistic △*K* based on comparing the log probability of the data (LnP(D)) for each value of *K* as described by Evanno et al.[39], and was implemented in the freely accessible STRUCTURE HARVESTER [40]. The highest △*K* value was selected to determine the number of clusters. The software CLUMPP v1.1.2 [41] was used to calculate the average membership coefficient (*Q*) for each individual, permute population membership coefficients matrices (*Q*-matrices) from 10 replicate cluster analyses and outputs a mean of the permuted matrices across replicates. Each individual’s probability of assignment to each cluster was visualized by Distruct software version 1.1 [42]. The membership coefficients of each individual also denote the proportion of an individual’s genome that originated in each cluster [38]. Individuals were assigned to each cluster with *Q* ≥ 0.6. Individuals with *Q* < 0.6 in each cluster were considered admixed, as suggested by previous studies [39, 43, 44]. The percentages of the *K* genetic pools from each population were displayed as pie charts, which were visualized with a partial map of Guangxi.

We used STRUCTURE 2.3 to identify the potential migrants between populations. The GENSBACK was set to 2, which will test each individual for evidence of ancestry from any of the six populations for two generations before the present with the MIGRPRIOR = 0.05. We used BOTTLENECK v.1.2.02 software [45] to determine whether the populations had recently experienced a bottleneck, assuming that the allelic diversity is reduced faster than the heterozygosity in a recently bottlenecked population. As a result, the observed heterozygosity (*He*) would be larger than the expected heterozygosity (*Heq*). We used the infinite alleles model (IAM) as the most appropriate evolutionary model for this study, with a sign test for each population and the entire range of *S*. *alterniflora* in Guangxi.

### Genetic, phenotypic and environmental/geographical associations

In 2013, five phenotypic traits data of *S*. *alterniflora* in population 1-BJ, 4-QS, and 5-XC were evaluated at the same time of sample collection (S1 Table): (1) the average fresh weight per plant (FW, g), (2) the average dry weight per plant (DW, g), (3) the average height (H, cm), (4) the average basal diameter (BD, mm), (5) the average number of nodes of a stem (N). Soil pH values and soil salinities of population 1-BJ, 4-QS, and 5-XC were also detected. Detailed methods and values of these phenotypic and environmental data for population 1-BJ, 4-QS, and 5-XC were reported previously by Zhao et al. [31] (S2 Table). The average annual mean temperature (T, °C) and the average annual precipitation (P, mm) of ten years (2006–2015) of the three populations in Guangxi were obtained from https://data.cma.cn/ (S2 Table). ANOVA analysis was performed to compare each of the phenotypic traits for *S*. *alterniflora* among population 1-BJ, 4-QS, and 5-XC.

The associations between genetic (GD), phenotypic (PD), and geographic distances among populations were tested by using the Mantel test in GENALEX 6.503 [36, 46]. The pairwise distance matrices of phenotypes differentiation were obtained by the measure of ‘dist’ in R package after scaled the phenotypic traits.

A principal coordinate analysis (PCOA) of the genetic data was performed with the pairwise genetic distance matrix by GENALEX 6.503 [36]. We also assessed whether the genetic differentiation (scores from four axes from the PCOA analysis of the SNP data) and phenotypic traits was influenced by environmental/geographical variables (soil salinities, soil pH, the average annual mean temperature, the average annual precipitation, longitude, latitude, and total variables) by using a redundancy analysis (RDA) within the ‘vegan’ package (v.2.3–1) [47]. The percentage contribution of this explained variation to each or the total environmental variation among the genetic or morphological groups was estimated, and the significance of the RDA results were tested with a global permutation (999 permutations).

## Results

### Population genetic diversity

The 176 individuals of *S*. *alterniflora* produced 372 AFLP fragments, of which 348 (93.55%) were polymorphic. In each population, 310 (5-XC) to 322 (1-BJ) polymorphic fragments were identified, with the percentage of polymorphic bands (*PPB*) varying from 83.33% (5-XC) to 86.56% (1-BJ) (Table 1). Considering all populations, Nei’s gene diversity (*H*) was 0.115, and Shannon’s information index (*I*) was 0.221. There was no obvious difference between the three genetic diversity indexes among the six populations (*P*-value = 1.000). 1-BJ had the highest observed number of alleles (*Na* = 1.867) and the highest effective number of alleles (*Ne* = 1.160). *H* and *I* were the highest in 1-BJ (*H* = 0.127, *I* = 0.232), lowest in 5-XC (*H* = 0.101, *I* = 0.192), and intermediate in 6-DW (*H* = 0.116, *I* = 0.215). The number of private loci (*P*_*L*_) was the highest in population 5-XC (*P*_*L*_ = 4), followed by 2-DS and 6-DW (*P*_*L*_ = 2), whereas no private loci were detected in the populations 3-ST and 4-QS. Compared the genetic diversity between our study and the study of Utomo et al. [9], the average heterozygosities (*H*) of *S*. *alterniflora* in Guangxi, China were significantly lower than *S*. *alterniflora* in undisturbed sites of Louisiana basins, USA (*P*-value = 0.000). The population bottleneck sign test showed the number of loci with a heterozygosity excess was greater than the number of loci with a heterozygosity deficiency in population 1-BJ, 2-DS, and 3-ST (Table 1). Five populations (1-BJ, 2-DS, 3-ST, 4-QS, and 6-BH) and the entire range of *S*. *alterniflora* in Guangxi have identified bottleneck signatures (*P*-value < 0.05).

### Genetic differentiation among populations

**Fig 2:**
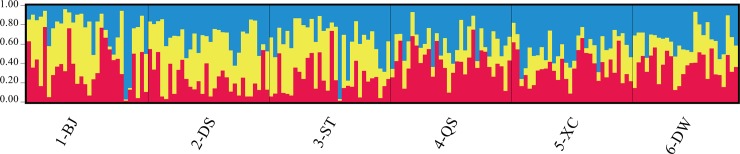
Bayesian assignment proportions for *K* = 3 genetic clusters determined in STRUCTURE, three clusters were represented by red, blue and yellow respectively.

Based on the STRUCTURE analysis, the highest △*K* value was observed at *K* = 3 (S1 Fig), indicating that the samples could be sorted into three genetic clusters, represented by three colors (red, blue and yellow) in Fig 2. However, the three genetic clusters did not correspond to our geographic sampling. A weak population structure in *S*. *alterniflora* was revealed. Only 44 (25%) individuals from the six populations could be assigned to the three clusters with a membership coefficient (*Q*) more than 0.6, and 132 individuals were genetic admixed (Table 2, Fig 2). Among them, there was no individual in 2-DS could be assigned to the red cluster, and no individual in 4-QS, 5-XC, and 6-DW could be assigned to the yellow cluster. Sixteen individuals in 1-BJ were genetically admixed, and the proportion was the lowest (53%). Higher proportions of admixed individuals were detected in other populations: 73% in 2-DS and 3-ST, and 77% in 4-QS and 5-XC. All samples in 6-DW were admixed individuals (100%).

**Table 2:** The number of individuals with *Q* ≥ 0.6 to each cluster (three clusters were represented by red, blue and yellow respectively) in the six populations and the number of admixed individuals in each population of *Spartina alterniflora*.

Pop. code	Sample size	No. of individuals			No. of admixed individuals	Proportion (%)
		Yellow	Blue	Red		
1-BJ	30	7 7 3 0 0 0	2 1 4 3 4 0	5 0 1 4 3 0	16	53.3
2-DS	30				22	73.3
3-ST	30				22	73.3
4-QS	30				23	76.6
5-XC	30				23	76.6
6-DW	26				26	100.0
Total	176	17	14	13	132	75.0

Admixed individual: *Q* values of the individual in three genetic clusters were all less than 0.6.

**Fig 3:**
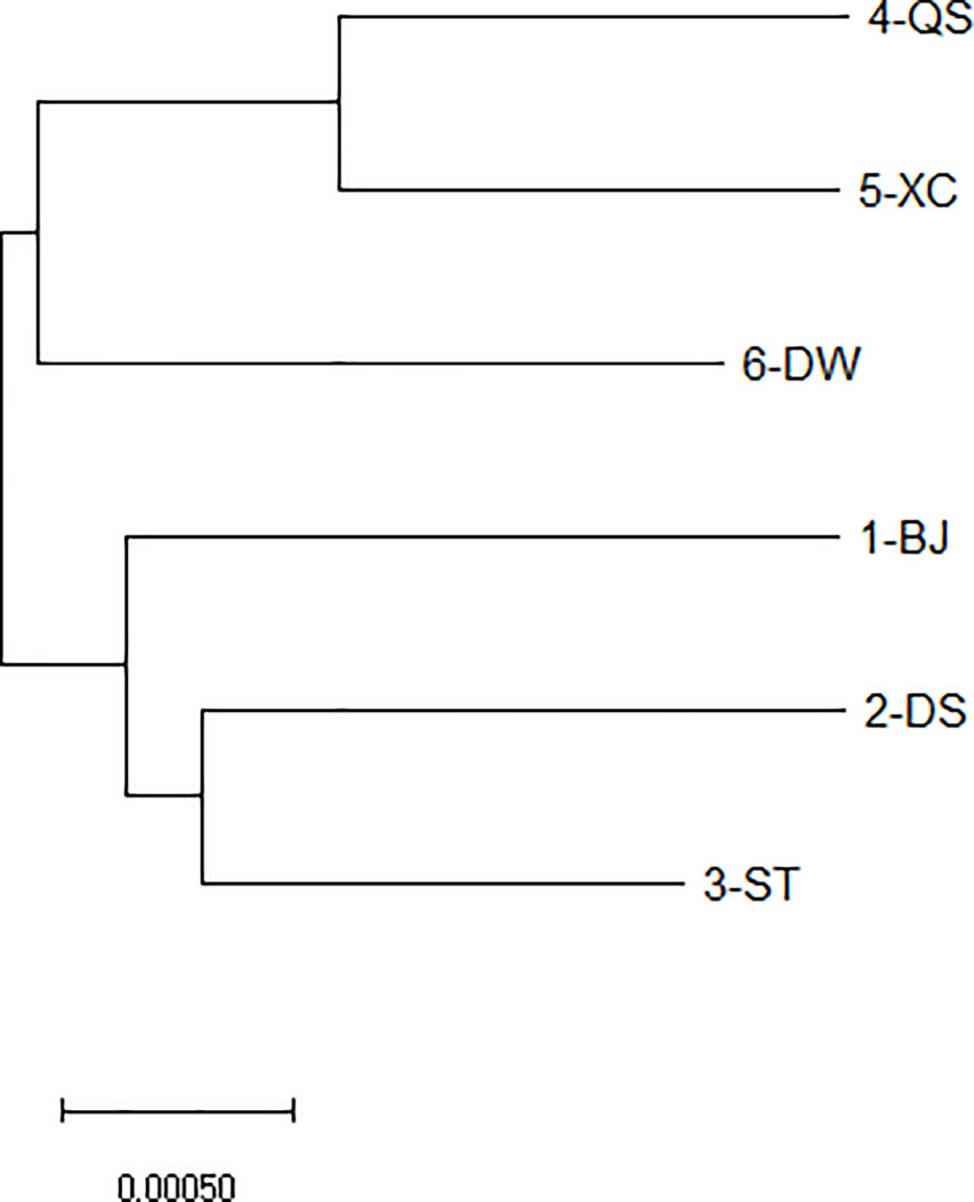
The dendrogram of the six *Spartina alterniflora* populations in Guangxi based on genetic distance.

AMOVA revealed that less than 1% variation occurred among populations and region, and 99% variation was within populations (Table 3). Genetic distance (*D*) between any two populations varied from 0.0022 to 0.0038, with an average distance of 0.0032. The closest genetic distance was observed between 4-QS and 5-XC (D = 0.0022), while the largest distance was observed between 4-QS and 2-DS (D = 0.0038) (Table 4). The six populations were grouped into three clades, with 1-BJ, 2-DS, and 3-ST forming the first clade, 4-QS and 5-XC forming the second clade, and 6-DW forming the third clade (Fig 3).

**Table 3:** Analysis of molecular variance (AMOVA) of the six *Spartina alterniflora* populations in Guangxi, China.

Source	df	Sum of squares	MS	Est. Var.	Variance (%)
Among regions	1	71.55	71.55	0.32	0.85
Among populations	4	246.06	49.21	0.42	0.61
Within populations	170	6283.97	36.97	36.97	98.54
Total	175	6530.03		37.38	100

**Table 4:** Nei's genetic identity (above diagonal) and genetic distance (below diagonal) of the six populations of *Spartina alterniflora* in Guangxi.

Pop. code	1-BJ	2-DS	3-ST	4-QS	5-XC	6-DW
1-BJ	—	0.9970	0.9971	0.9963	0.9962	0.9967
2-DS	0.0030	—	0.9976	0.9961	0.9962	0.9966
3-ST	0.0029	0.0024	—	0.9971	0.9969	0.9969
4-QS	0.0037	0.0040	0.0029	—	0.9978	0.9967
5-XC	0.0038	0.0038	0.0031	0.0022	—	0.9968
6-DW	0.0033	0.0034	0.0032	0.0033	0.0032	—

### Detection of contemporary migration and gene flow

**Fig 4:**
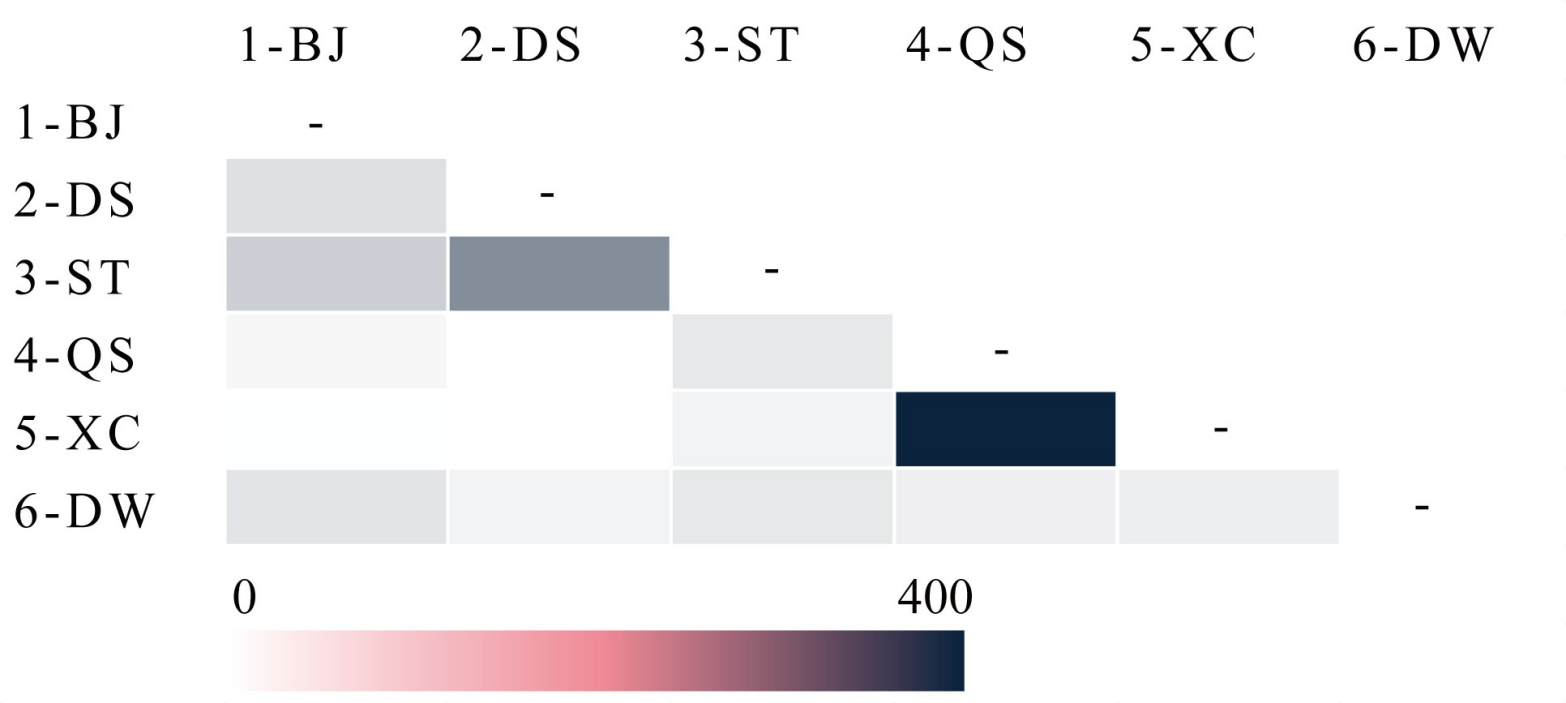
Contemporary gene flow among populations of *Spartina alterniflora* in Guangxi, the color shades represented levels of gene flow.

The mean value of pairwise genetic differentiation (*Φpt*) among all populations was 0.015. The gene flows of the six populations are provided as a heat map (Fig 4). High levels of gene flow were found between populations, ranged from 21.540 (2-DS and 5-XC) to 363.207 (4-QS and 5-XC). Assignment tests identified 41 individuals with at least a 95% probability of belonging to their own predefined geographic population and eight individuals above 90%. Fifty-six individuals showed evidence of an ancestor having immigrated from another location within three generations, with the immigration probabilities more than 0.50 (Table 5, S3 Table). The number of migrants between a population and its adjacent populations (35) was obviously larger than that between the population and its non-adjacent populations (21) (considering that the distance between 2-DS and 3-ST was very small, both of them were taken as adjacent populations of 1-BJ and 4-QS in this analysis).

**Table 5:** The number of migrants among populations of *Spartina alterniflora* in Guangxi based on the inference of recent migration rates from Structure assignments.

Pop. code	1-BJ	2-DS	3-ST	4-QS	5-XC	6-DW
1-BJ	-	**4**	**2**	2	2	1
2-DS	**1**	-	**0**	**1**	0	0
3-ST	**5**	**4**	-	**1**	4	0
4-QS	1	**0**	**3**	-	**1**	2
5-XC	1	2	1	**5**	-	**8**
6-DW	3	1	1	**0**	**0**	-

The numbers of migrants between adjacent populations are highlighted in bolds.

### Genetic, phenotypic and environmental/geographical associations

The five phenotypic traits between population 1-BJ, 4-QS, and 5-XC were significant differences (S2 Fig). *S*. *alterniflora* in Qingshantou Village (4-QS) was obvious higher and stouter than in Beijie Village (1-BJ) and Xicungang harbor (5-XC). Positive correlation between genetic distances and geographic distances (*r* = 0.53, *P*-value = 0.02) and between genetic distances and shoreline distances (*r* = 0.62, *P*-value = 0.04) were found among six populations (S3 Fig). However, there was no evidence for a relationship between genetic (GD) and phenotypic (PD) distances (*r* = -1.00, *P*-value = 0.18) and between phenotypic (PD) distances and geographic distances of population 1-BJ, 4-QS and 5-XC (*r* = -0.97, *P*-value = 0.40) (S4 Fig).

**Fig 5:**
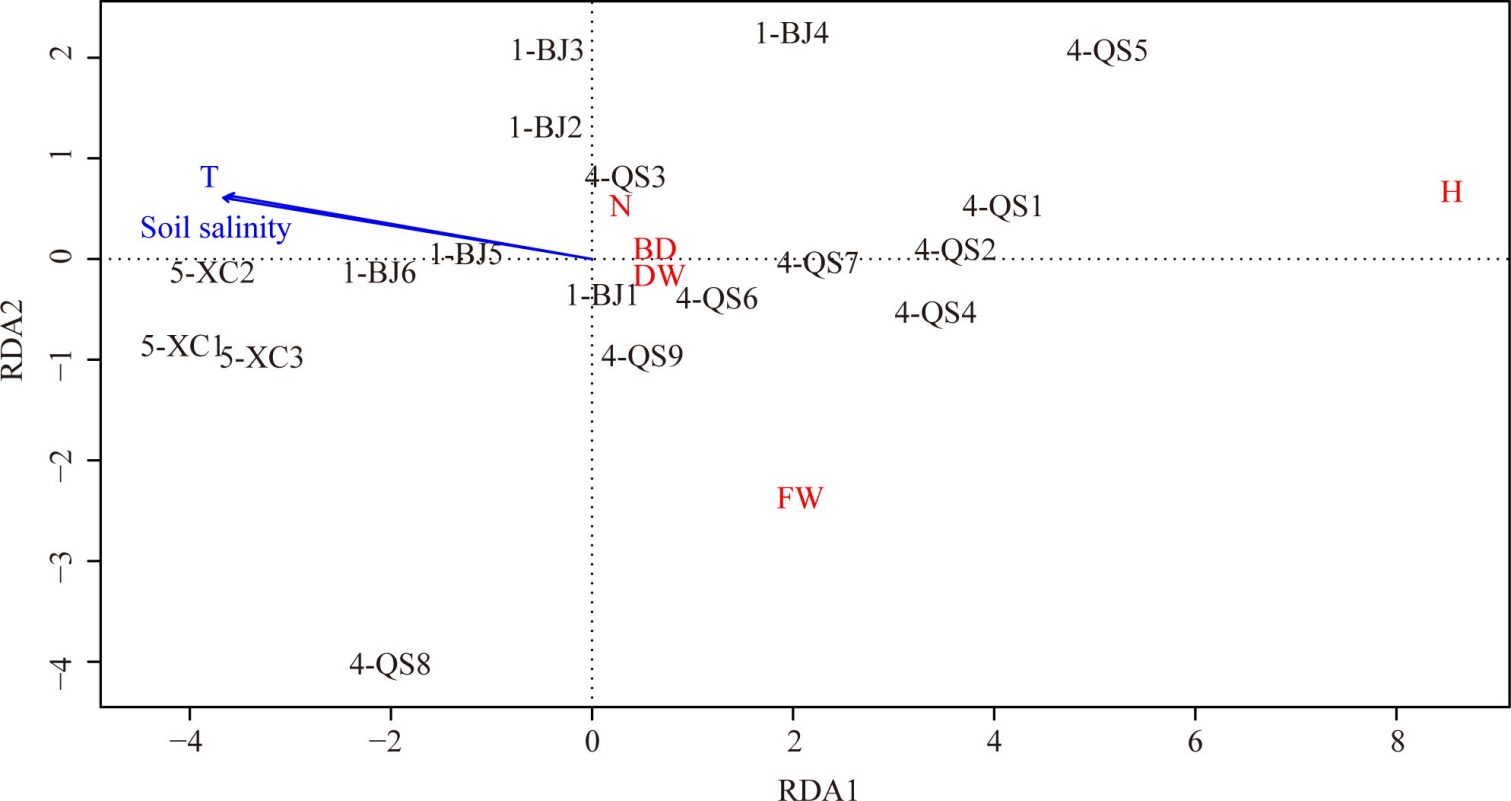
Ordination diagram of redundancy analysis (RDA) with quadrats, phenotypic traits, and environmental variables (arrows) of population 1-BJ, 4-QS, and 5-XC. FW: the average fresh weight per plant; DW: the average dry weight per plant, H: the average height; BD: the average basal diameter; N: the average number of nodes of a stem; T: the average annual mean temperature.

The RDA analysis revealed that 59.37% of the phenotypic variable for population 1-BJ, 4-QS, and 5-XC was explained by the first one axes. Axis 1 was significantly negatively correlated with soil salinities (43.85% explained of the total variable, *P*-value = 0.001) and the average annual mean temperature (44.99% explained of the total variable, *P*-value = 0.003) (Table 6, Fig 5). Only 3.064% of the genetic differentiation was explained by environmental variables. The RDA results suggested that the environmental variables account for phenotypic differences but not for the genetic differentiation.

**Table 6:** A redundancy analysis (RDA) of the phenotypic and environmental/geographic variables in population 1-BJ, 4-QS, and 5-XC.

Environmental/ Geographic	Constrained	% Explained	*F* value	Prob > *F*
Soil salinity	271.89	43.85	12.497	0.001
T (°C)	278.92	44.99	13.085	0.003
P (mm)	21.156	3.412	0.5653	0.459
Soil pH	118.57	19.12	3.784	0.072
Latitude (N)	7.599	1.226	0.198	0.654
Longitude (E)	8.748	1.411	0.23	0.625
Total	370.8	59.81	11.162	0.002

T: the average annual mean temperature; P: the average annual precipitation.

## Discussion

### Low genetic diversity and weak genetic structure contrasts with high phenotypic variability

Genetic bottlenecks were detected in five of the six populations and the entire range of *S*. *alterniflora* in Guangxi, China. As genetic bottlenecks could result in decreased genetic diversity, low genetic diversity of *S*. *alterniflora* in Guangxi was not unexpected. Similar situations have also been revealed in other invasive regions of *S*. *alterniflora* in China [13, 23, 24]. However, by comparison of genetic diversity of the six populations, we observed no obvious decline of genetic diversity in the younger populations (4-QS, 5-XC, and 6-DW) of *S*. *alterniflora*. The newly-established population (6-DW) had similar genetic diversity index as the oldest population (1-BJ). These findings indicate that the low genetic diversity of *S*. *alterniflora* in Guangxi would not become an obstacle for population establishment and expansion. Moreover, genetic diversity could be successfully maintained at a certain level during the dispersal process of *S*. *alterniflora*.

The six populations of *S*. *alterniflora* in Guangxi showed very low genetic differentiation (*Φpt* = 0.015). We also found that more than 99% of genetic variance was distributed within populations, whereas only 1% was between regions and populations. Despite high within-population genetic diversity was consistent with prior studies, the proportion of genetic variation among populations of *S*. *alterniflora* in its native range (96.59%, Louisiana, USA) [9] and in restored ranges (85%, New York, USA) [48] were all higher than in Guangxi, China. This is likely due to high gene flows between populations in Guangxi. Another possibility is that the short invasion time is insufficient to result in significant genetic differentiation among the six populations of *S*. *alterniflora*. The low population differentiation between the two regions also confirmed that the Dandou Sea was not a partial barrier to gene flow between the earlier-established (1-BJ, 2-DS and 3-ST) and the later-established populations (4-QS, 5-XC and 6-DW).

High levels of phenotypic variability among these genetically similar *S*. *alterniflora* populations and no correlativity between phenotypic differences and genetic differentiation might provide evidence of phenotypic plasticity. Phenotypic variation of *S*. *alterniflora* in China due to phenotypic plasticity rather than genetic variation was also revealed in the study of Liu et al. by a common garden experiment [49]. Low genetic diversity with high phenotypic variability was also revealed in other species of *Spartina*, such as *S*. *densiflora* populations in North America [50]. Therefore, in spite of genetic bottlenecks and low genetic diversity might be the negative effects for invasive alien species spreading, high phenotypic plasticity may improve their responses to new environments [29, 51, 52]

### Single introduction and natural dispersal were revealed in Guangxi *Spartina alterniflora* populations

A significant correlation between genetic distances and geographic distances was revealed in this study. Three clades in NJ dendrogram based on genetic distances between populations were also in accordance with the geographical distribution of the populations. The ages of the six populations and geographic distances among them were purely coincidental (geographic distance between the oldest populations of 1-BJ, 2-DS and 3-ST and the youngest population of 6-DW was the farthest, and the middle-aged populations of 4-QS and 5-XC were located between 1-BJ, 2-DS, 3-ST, and 6-DW). This result implied that *S*. *alterniflora* populations in Guangxi most likely to be the result of a single introduction in Beijie from the Dandou Sea coast in 1994, and spread has occurred via natural pathways. This might be one reason why there were genetic bottlenecks and low genetic diversities in *S*. *alterniflora* populations in Guangxi, on account of multiple introductions from different sources could help invasive species to maintain a high level of genetic variation [53, 54].

In addition, higher gene flows and more migrations were detected in adjacent populations than in non-adjacent populations implied the natural dispersal of *S*. *alterniflora* in Guangxi either. Although Liu et al. found *S*. *alterniflora* at low latitudes in China had low sexual reproductive activity [49], the migrations between populations were considered as evidence for the potential for waterborne long-distance dispersal of seeds [48, 55–57].

### *Spartina alterniflora* in Guangxi came from a pre-admixed population

The three genetic clusters revealed by STRUCTURE analysis concurred with previous findings that *S*. *alterniflora* in China originated from the three ecotypes in Florida, Georgia, and North Carolina. Due to a lack of samples from the native region in this study, we could not confirm whether there was a one-to-one correspondence between the three clusters and the three ecotypes. We found high proportions of admixture individuals in all of the six populations even the early introduced site—Beijie Village (1-BJ), agreeing with previous studies showing that the hybrid and mixture of ecotypes coexist in China, especially in southern populations [13, 24]. It is most likely that genetic admixture occurred before *S*. *alterniflora* colonized in Guangxi [13]. We also found that admixture increased when *S*. *alterniflora* spread to a new area in Guangxi. The proportion of admixed individuals in the newly-established population of 6-DW (100%) was much higher than that in the earliest population of 1-BJ (53%).

The admixture was revealed as the most important factors for successful invasion of invasive species [2, 13, 21, 24]. However, we can’t compare the effect on successful invasion between pre-introduction admixture and post-introduction admixture [58]. If pre-introduction admixture had similar benefits as post-introduction admixture, such heterogeneous mixture might increase genetic or phenotype novelty rather than genetic diversity [2]. The novel genotypes or phenotypes could increase fitness to diverse ecological spaces and assist in extending the species range [2, 59].

### Associations between phenotypic variation, genetic differentiation, and the environment

The genetic differentiation between population 1-BJ, 4-QS, and 5-XC was not influenced by the four environmental variables. However, we found the phenotypic variation of *S*. *alterniflora* was obviously impacted by soil salinities and the average annual mean temperature of these sites. Previous studies have also shown those plant traits of *S*. *alterniflora* such as height and biomass could respond to temperature change [31, 60, 61]. Salinity inhibited the growth of *S*. *alterniflora* have been reported in studies of Nestler [62] and Huang [63]. Height, basal diameter, and leaf area decrease with the rise of salinity [63]. Liu et al. [49] and Zhao et al. [31] reported the phenotypic traits of *S*. *alterniflora* along the east coast of China exhibited linear relationships with latitude whereas our study did not support this inference due to population 1-BJ, 4-QS and 5-XC distributed in a small scale range. However, the study of Zhao et al. [31] didn’t find the association between phenotypic variation and soil salinities. It might be because broad phenotypic traits differences which were impacted by latitude in a large scale range would mask some small impact factors.

## Conclusions

In Guangxi, we found obvious genetic bottlenecks, low genetic diversity, and low levels of genetic differentiation among populations of *S*. *alterniflora*. However, high phenotypic variability influenced by environmental factors was also revealed. Therefore, we conclude that although *S*. *alterniflora* invaded into Guangxi by a single introduction from a pre-admixed population. High phenotypic plasticity would help this alien species overcome negative effect and colonized in a wide variety of environment. In spite of the previous study suspected *S*. *alterniflora* populations at low latitudes may spread slower [49], the new invaded site was still being found. As the Dandou Sea coast is the main source for the spread of *S*. *alterniflora* in Guangxi, the population in this area should also be strictly monitored and managed to avoid its further spread.

## Supporting information

S1 Fig *Spartina alterniflora* plots of the log-likelihood, ln P(D) (a), for ten runs at each value of K, and the second-order rate of change in ln P(D) (b), ΔK, as a function of the number of clusters, K, from the analyses of all samples.
(TIF)

S2 Fig Box plots of the average fresh weight per plant (FW, g), the average dry weight per plant (DW, g), the average height (H, cm), the average basal diameter (BD, mm), and the average number of nodes of a stem (N). Different letters indicate significant differences (*P*-value < 0.05, ANOVA) between population 1-BJ, 4-QS, and 5-XC.
(TIF)

S3 Fig The correlations of genetic distances with geographic distances (A) and shoreline distances (B) by the Mantel test.
(TIF)

S4 Fig The correlations of phenotypic distances (PD) with genetic (GD) (A) and geographic distances (B) by the Mantel test.
(TIF)

S1 Table The five phenotypic traits data of *Spartina alterniflora* in population 1-BJ, 4-QS and 5-XC from the study of Zhao et al. [31]. (DOCX)

S2 Table The environmental information of population 1-BJ, 4-QS, and 5-XC. (DOCX)

S3 Table The inference of recent migration rates from Structure assignments. (DOCX)
