# Supplementary figures and images for: Low genetic differentiation yet high phenotypic variation in the invasive populations of Spartina alterniflora in Guangxi, China
Source: PLoS One. 2019 Sep 17;14(9):e0222646. doi: 10.1371/journal.pone.0222646 (PMC6748429; doi:10.1371/journal.pone.0222646)

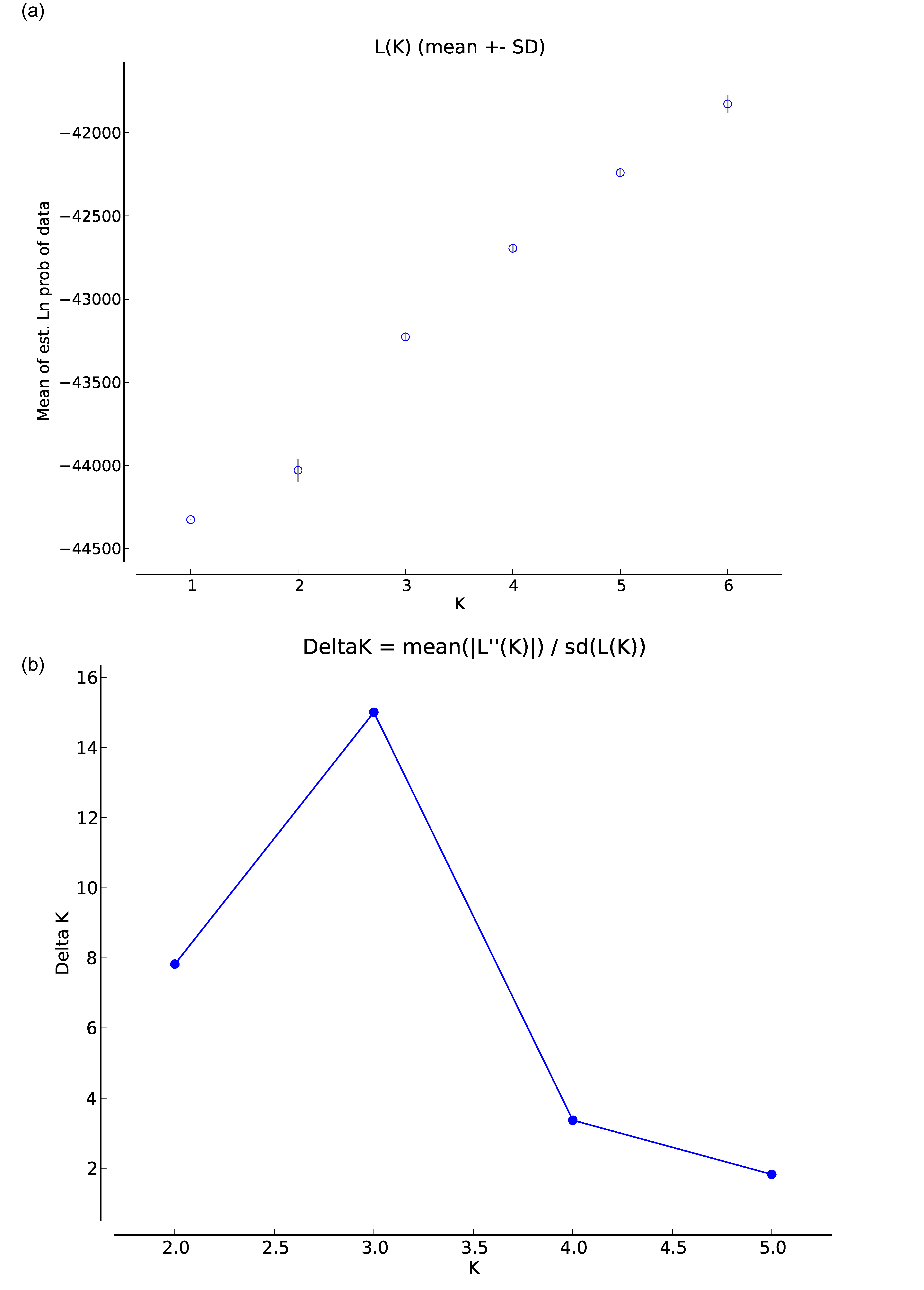

Supplement: S1 Fig — Spartina alterniflora plots of the log-likelihood, ln P(D) (a), for ten runs at each value of K, and the second-order rate of change in ln P(D) (b), ΔK, as a function of the number of clusters, K, from the analyses of all samples. (TIF) [file pone.0222646.s001.tif]

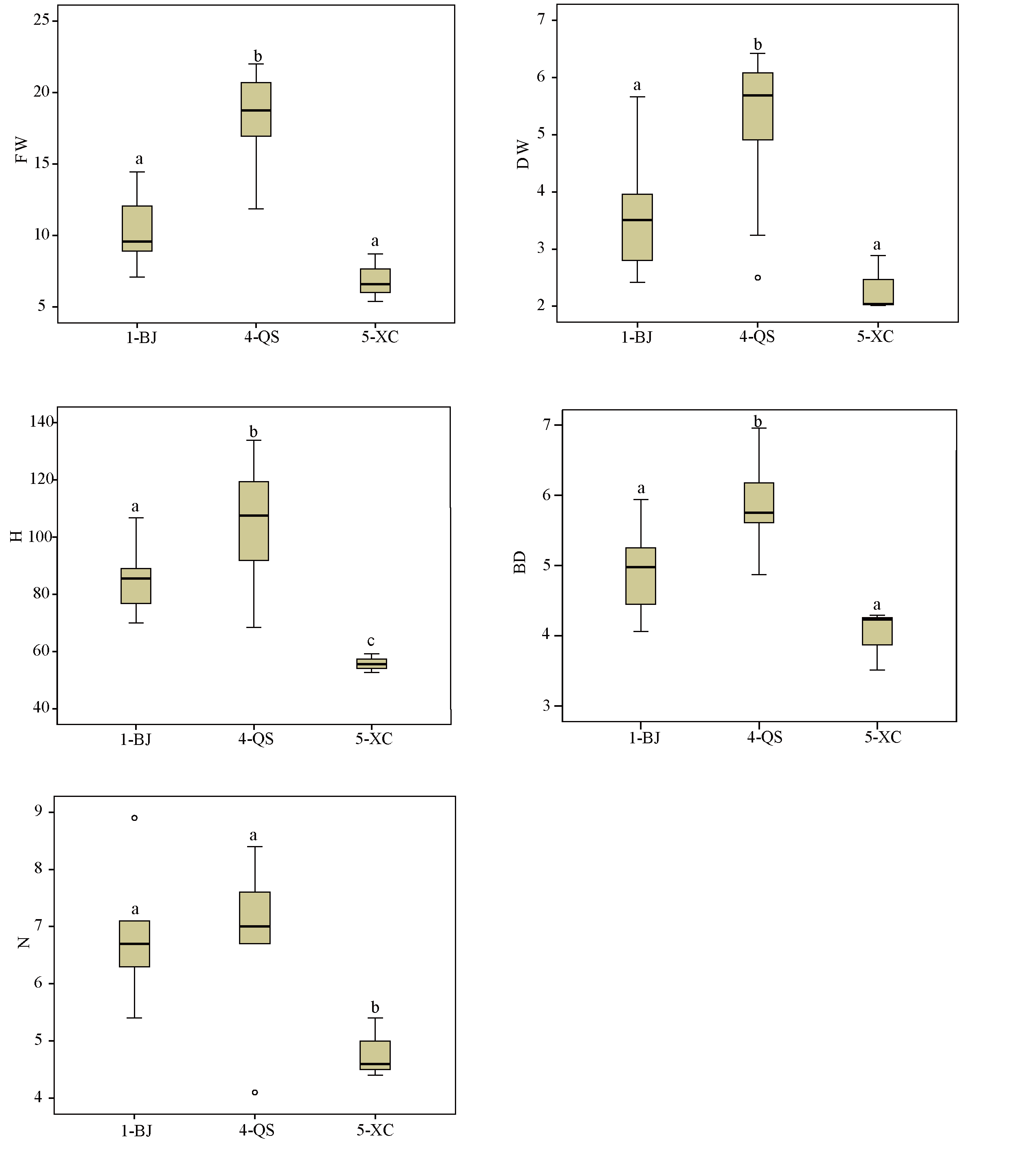

Supplement: S2 Fig — Different letters indicate significant differences (P-value < 0.05, ANOVA) between population 1-BJ, 4-QS, and 5-XC. (TIF) [file pone.0222646.s002.tif]

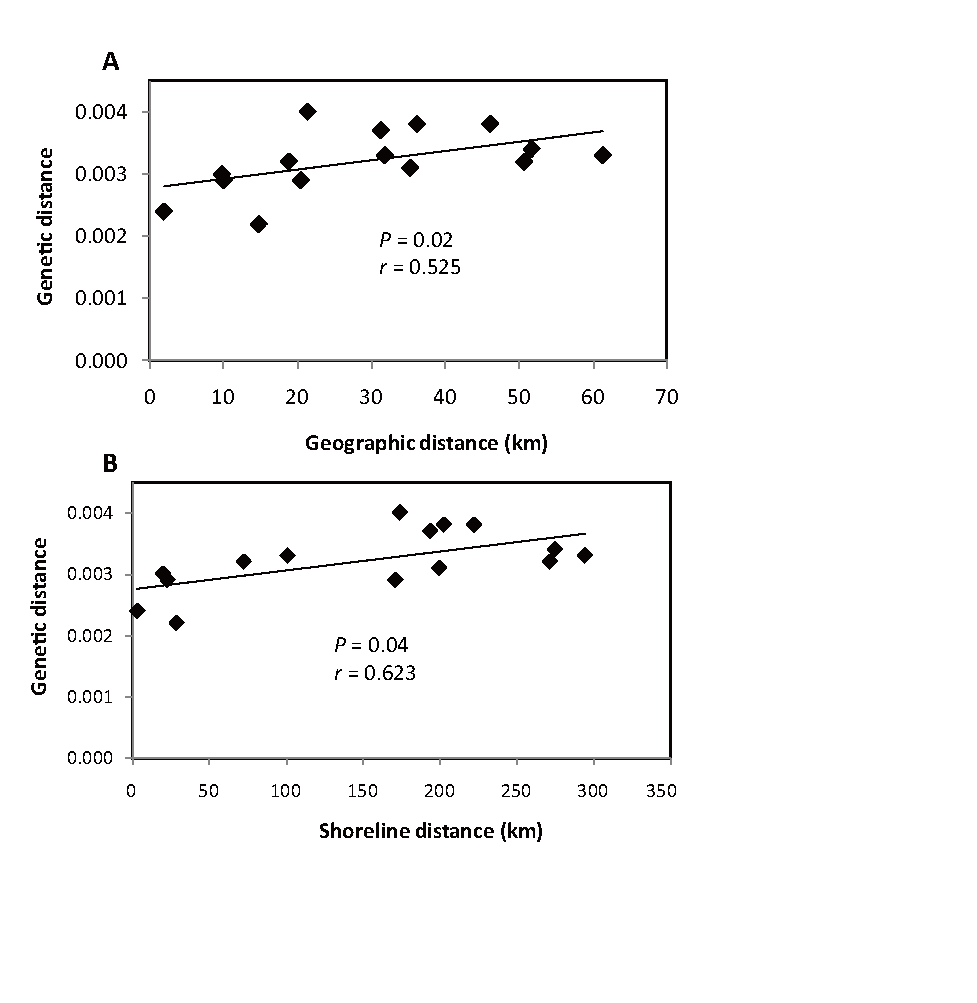

Supplement: S3 Fig — The correlations of genetic distances with geographic distances (A) and shoreline distances (B) by the Mantel test. (TIF) [file pone.0222646.s003.tif]

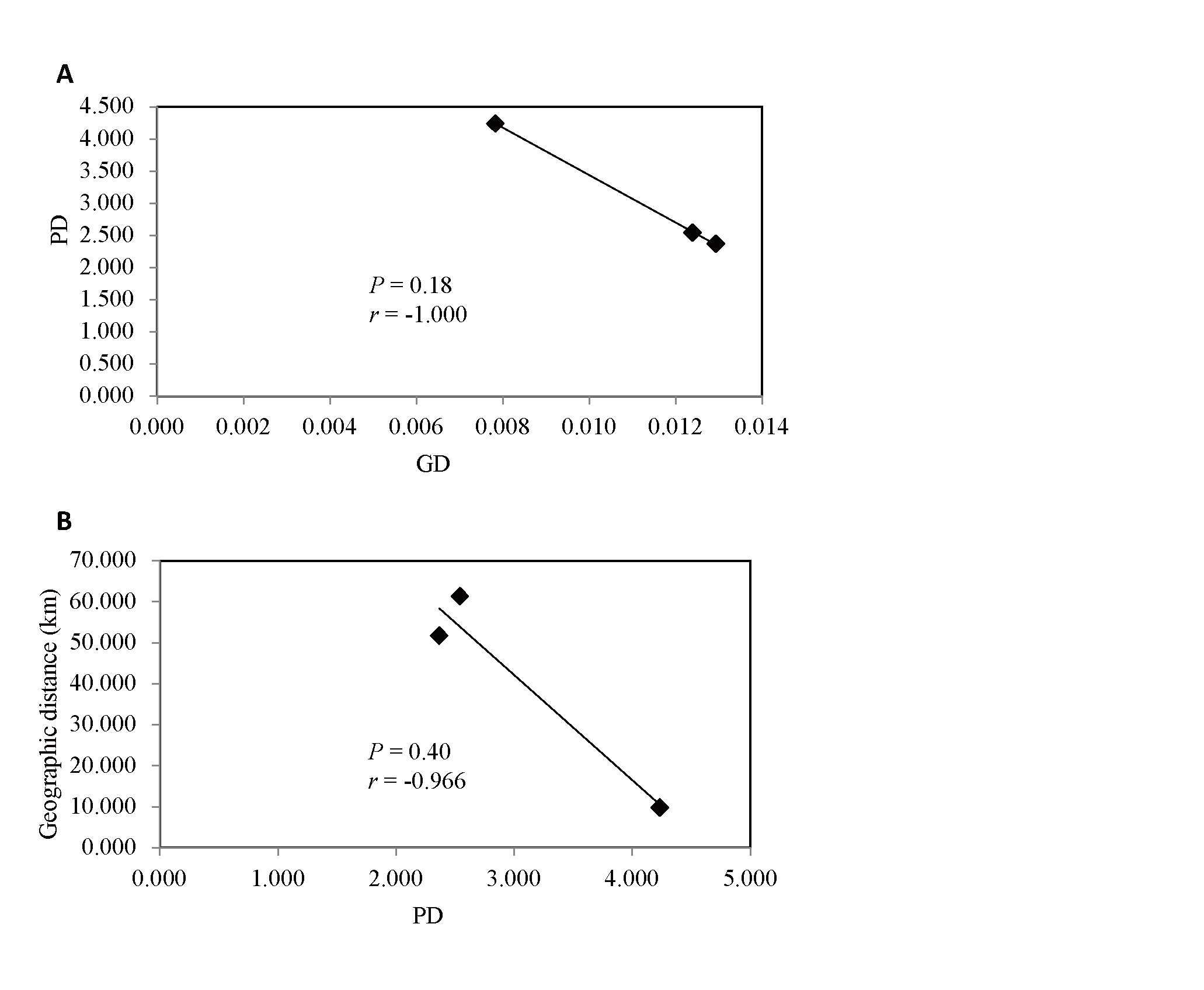

Supplement: S4 Fig — The correlations of phenotypic distances (PD) with genetic (GD) (A) and geographic distances (B) by the Mantel test. (TIF) [file pone.0222646.s004.tif]
