# Supplementary material for: Low genetic differentiation yet high phenotypic variation in the invasive populations of Spartina alterniflora in Guangxi, China
Source: PLoS One. 2019 Sep 17;14(9):e0222646. doi: 10.1371/journal.pone.0222646 (PMC6748429; doi:10.1371/journal.pone.0222646)
Supplement: S1 Table — (DOCX) [file pone.0222646.s005.docx]

**S1 Table. The five phenotypic traits data of *Spartina alterniﬂora* in population 1-BJ, 4-QS and 5-XC from the study of Zhao et al. [**[**31**](#_ENREF_31)**]**

| Quadrat | FW(g) | DW(g) | H(cm) | BD(mm) | N |
| --- | --- | --- | --- | --- | --- |
| 1-BJ1 | 14.45 | 3.96 | 89 | 5.94 | 6.6 |
| 1-BJ2 | 8.9 | 3.67 | 85.2 | 5.06 | 6.3 |
| 1-BJ3 | 7.1 | 3.35 | 85.87 | 4.9 | 6.8 |
| 1-BJ4 | 12.05 | 5.66 | 106.72 | 5.25 | 8.9 |
| 1-BJ5 | 9.9 | 2.8 | 76.8 | 4.45 | 5.4 |
| 1-BJ6 | 9.25 | 2.42 | 70 | 4.06 | 7.1 |
| 4-QS1 | 20.75 | 6.08 | 123.7 | 6.96 | 8.4 |
| 4-QS2 | 20.7 | 5.99 | 119.36 | 6.74 | 7.6 |
| 4-QS3 | 11.85 | 4.91 | 91.58 | 4.87 | 6.8 |
| 4-QS4 | 22 | 5.31 | 117.33 | 5.14 | 7.33 |
| 4-QS5 | 19.25 | 2.5 | 133.86 | 5.75 | 7.9 |
| 4-QS6 | 16.7 | 6.42 | 98.6 | 5.92 | 6.7 |
| 4-QS7 | 18 | 6.15 | 107.49 | 6.18 | 7 |
| 4-QS8 | 18.75 | 5.69 | 68.37 | 5.61 | 4.1 |
| 4-QS9 | 16.95 | 3.24 | 91.9 | 5.63 | 6.7 |
| 5-XC1 | 6.6 | 2.01 | 52.6 | 4.23 | 4.4 |
| 5-XC2 | 5.4 | 2.04 | 55.6 | 3.51 | 4.6 |
| 5-XC3 | 8.7 | 2.89 | 59.2 | 4.29 | 5.4 |

FW: the average fresh weight per plant; DW: the average dry weight per plant, H: the average height; BD: the average basal diameter; N: the average number of nodes of a stem.
