# Supplementary material for: Low genetic differentiation yet high phenotypic variation in the invasive populations of Spartina alterniflora in Guangxi, China
Source: PLoS One. 2019 Sep 17;14(9):e0222646. doi: 10.1371/journal.pone.0222646 (PMC6748429; doi:10.1371/journal.pone.0222646)
Supplement: S2 Table — (DOCX) [file pone.0222646.s006.docx]

**S2 Table. The environmental information of population 1-BJ, 4-QS, and 5-XC**

| Pop. | Soil pH | Soil salinity | T (°C) | P (mm) |
| --- | --- | --- | --- | --- |
| 1-BJ | 6.88 | 28.30 | 23.23 | 1733.72 |
| 4-QS | 7.01 | 22.70 | 23.11 | 1795.62 |
| 5-XC | 7.30 | 28.50 | 23.23 | 1843.11 |

T: the average annual mean temperature; P: the average annual precipitation.
