# Supplementary material for: Low genetic differentiation yet high phenotypic variation in the invasive populations of Spartina alterniflora in Guangxi, China
Source: PLoS One. 2019 Sep 17;14(9):e0222646. doi: 10.1371/journal.pone.0222646 (PMC6748429; doi:10.1371/journal.pone.0222646)
Supplement: S3 Table — (DOCX) [file pone.0222646.s007.docx]

**S3 Table. The inference of recent migration rates from Structure assignments.**

| Ind. | Predefined population | 1-BJ | | | 2-DS | | | 3-ST | | | 4-QS | | | 5-XC | | | 6-DW | | |
| --- | --- | --- | --- | --- | --- | --- | --- | --- | --- | --- | --- | --- | --- | --- | --- | --- | --- | --- | --- |
|  |  | Pr(0) | Pr(1) | Pr(2) | Pr (0) | Pr (1) | Pr (2) | Pr (0) | Pr (1) | Pr (2) | Pr (0) | Pr (1) | Pr (2) | Pr (0) | Pr (1) | Pr (2) | Pr (0) | Pr (1) | Pr (2) |
| A4 | 1-BJ | 0.156 | -- | -- | ***0.592*** | ***0.000*** | ***0.002*** | 0.000 | 0.000 | 0.000 | 0.000 | 0.000 | 0.000 | 0.000 | 0.000 | 0.000 | 0.246 | 0.000 | 0.004 |
| A6 | 1-BJ | 0.298 | -- | -- | ***0.694*** | ***0.000*** | ***0.005*** | 0.000 | 0.000 | 0.000 | 0.000 | 0.000 | 0.000 | 0.000 | 0.000 | 0.000 | 0.002 | 0.000 | 0.001 |
| A7 | 1-BJ | 0.385 | -- | -- | 0.048 | 0.000 | 0.004 | ***0.538*** | ***0.000*** | ***0.005*** | 0.012 | 0.000 | 0.002 | 0.000 | 0.000 | 0.000 | 0.005 | 0.000 | 0.001 |
| A13 | 1-BJ | 0.422 | -- | -- | ***0.544*** | ***0.000*** | ***0.005*** | 0.028 | 0.000 | 0.001 | 0.000 | 0.000 | 0.000 | 0.000 | 0.000 | 0.000 | 0.000 | 0.000 | 0.000 |
| A17 | 1-BJ | 0.001 | -- | -- | ***0.859*** | ***0.000*** | ***0.000*** | 0.004 | 0.000 | 0.000 | 0.003 | 0.000 | 0.000 | 0.133 | 0.000 | 0.000 | 0.001 | 0.000 | 0.000 |
| A19 | 1-BJ | 0.009 | -- | -- | 0.000 | 0.000 | 0.000 | 0.000 | 0.000 | 0.000 | 0.000 | 0.000 | 0.000 | 0.000 | 0.000 | 0.000 | ***0.990*** | ***0.000*** | ***0.001*** |
| A21 | 1-BJ | 0.443 | -- | -- | 0.000 | 0.000 | 0.000 | 0.000 | 0.000 | 0.000 | ***0.510*** | ***0.000*** | ***0.004*** | 0.009 | 0.000 | 0.011 | 0.019 | 0.000 | 0.004 |
| A25 | 1-BJ | 0.000 | -- | -- | 0.000 | 0.000 | 0.000 | 0.000 | 0.000 | 0.000 | 0.000 | 0.000 | 0.000 | ***1.000*** | ***0.000*** | ***0.000*** | 0.000 | 0.000 | 0.000 |
| A26 | 1-BJ | 0.000 | -- | -- | 0.000 | 0.000 | 0.000 | 0.000 | 0.000 | 0.000 | 0.003 | 0.000 | 0.000 | ***0.997*** | ***0.000*** | ***0.000*** | 0.000 | 0.000 | 0.000 |
| A28 | 1-BJ | 0.260 | -- | -- | 0.017 | 0.000 | 0.002 | ***0.717*** | ***0.000*** | ***0.003*** | 0.000 | 0.000 | 0.000 | 0.000 | 0.000 | 0.000 | 0.000 | 0.000 | 0.000 |
| A30 | 1-BJ | 0.239 | -- | -- | 0.080 | 0.000 | 0.002 | 0.012 | 0.000 | 0.001 | ***0.643*** | ***0.000*** | ***0.003*** | 0.007 | 0.000 | 0.012 | 0.000 | 0.000 | 0.000 |
| B24 | 2-DS | ***0.614*** | ***0.000*** | ***0.000*** | 0.010 | -- | -- | 0.375 | 0.000 | 0.001 | 0.000 | 0.000 | 0.000 | 0.000 | 0.000 | 0.000 | 0.000 | 0.000 | 0.000 |
| B29 | 2-DS | 0.139 | 0.000 | 0.001 | 0.174 | -- | -- | 0.000 | 0.000 | 0.000 | ***0.500*** | ***0.000*** | ***0.002*** | 0.001 | 0.000 | 0.002 | 0.182 | 0.000 | 0.000 |
| C5 | 3-ST | 0.002 | 0.000 | 0.000 | ***0.886*** | ***0.000*** | ***0.001*** | 0.111 | -- | -- | 0.000 | 0.000 | 0.000 | 0.000 | 0.000 | 0.000 | 0.000 | 0.000 | 0.000 |
| C9 | 3-ST | ***0.505*** | ***0.000*** | ***0.001*** | 0.025 | 0.000 | 0.002 | 0.456 | -- | -- | 0.000 | 0.000 | 0.000 | 0.000 | 0.000 | 0.000 | 0.010 | 0.000 | 0.002 |
| C10 | 3-ST | ***0.611*** | ***0.000*** | ***0.000*** | 0.155 | 0.000 | 0.000 | 0.000 | -- | -- | 0.234 | 0.000 | 0.000 | 0.000 | 0.000 | 0.000 | 0.000 | 0.000 | 0.000 |
| C12 | 3-ST | 0.219 | 0.000 | 0.001 | ***0.559*** | ***0.000*** | ***0.003*** | 0.217 | -- | -- | 0.000 | 0.000 | 0.000 | 0.000 | 0.000 | 0.000 | 0.000 | 0.000 | 0.000 |
| C13 | 3-ST | ***0.688*** | ***0.000*** | ***0.002*** | 0.004 | 0.000 | 0.001 | 0.114 | -- | -- | 0.175 | 0.000 | 0.000 | 0.000 | 0.000 | 0.000 | 0.015 | 0.000 | 0.001 |
| C16 | 3-ST | 0.299 | 0.000 | 0.000 | 0.000 | 0.000 | 0.000 | 0.000 | -- | -- | ***0.700*** | ***0.000*** | ***0.000*** | 0.000 | 0.000 | 0.000 | 0.000 | 0.000 | 0.000 |
| C17 | 3-ST | ***0.582*** | ***0.000*** | ***0.000*** | 0.234 | 0.000 | 0.000 | 0.043 | -- | -- | 0.139 | 0.000 | 0.000 | 0.000 | 0.000 | 0.000 | 0.001 | 0.000 | 0.000 |
| C18 | 3-ST | 0.000 | 0.000 | 0.000 | 0.000 | 0.000 | 0.000 | 0.000 | -- | -- | 0.000 | 0.000 | 0.000 | ***1.000*** | ***0.000*** | ***0.000*** | 0.000 | 0.000 | 0.000 |
| C19 | 3-ST | ***0.614*** | ***0.000*** | ***0.004*** | 0.020 | 0.000 | 0.001 | 0.361 | -- | -- | 0.000 | 0.000 | 0.000 | 0.000 | 0.000 | 0.000 | 0.000 | 0.000 | 0.000 |
| C20 | 3-ST | 0.000 | 0.000 | 0.000 | 0.000 | 0.000 | 0.000 | 0.000 | -- | -- | 0.069 | 0.000 | 0.000 | ***0.931*** | ***0.000*** | ***0.000*** | 0.000 | 0.000 | 0.000 |
| C25 | 3-ST | 0.204 | 0.000 | 0.000 | ***0.748*** | ***0.000*** | ***0.000*** | 0.048 | -- | -- | 0.000 | 0.000 | 0.000 | 0.000 | 0.000 | 0.000 | 0.000 | 0.000 | 0.000 |
| C27 | 3-ST | 0.001 | 0.000 | 0.001 | ***0.498*** | ***0.000*** | ***0.005*** | 0.469 | -- | -- | 0.000 | 0.000 | 0.001 | 0.000 | 0.000 | 0.006 | 0.018 | 0.000 | 0.001 |
| C28 | 3-ST | 0.000 | 0.000 | 0.000 | 0.117 | 0.000 | 0.002 | 0.346 | -- | -- | 0.000 | 0.000 | 0.001 | ***0.491*** | ***0.000*** | ***0.041*** | 0.000 | 0.000 | 0.001 |
| C29 | 3-ST | 0.000 | 0.000 | 0.000 | 0.007 | 0.000 | 0.001 | 0.209 | -- | -- | 0.173 | 0.000 | 0.001 | ***0.566*** | ***0.000*** | ***0.032*** | 0.010 | 0.000 | 0.001 |
| D2 | 4-QS | 0.010 | 0.000 | 0.000 | 0.000 | 0.000 | 0.000 | ***0.744*** | ***0.000*** | ***0.000*** | 0.001 | -- | -- | 0.000 | 0.000 | 0.000 | 0.244 | 0.000 | 0.000 |
| D6 | 4-QS | ***0.723*** | ***0.000*** | ***0.003*** | 0.000 | 0.000 | 0.000 | 0.000 | 0.000 | 0.000 | 0.273 | -- | -- | 0.000 | 0.000 | 0.000 | 0.000 | 0.000 | 0.000 |
| D7 | 4-QS | 0.006 | 0.000 | 0.000 | 0.000 | 0.000 | 0.000 | 0.000 | 0.000 | 0.000 | 0.005 | -- | -- | 0.000 | 0.000 | 0.000 | ***0.989*** | ***0.000*** | ***0.000*** |
| D11 | 4-QS | 0.000 | 0.000 | 0.000 | 0.000 | 0.000 | 0.000 | 0.000 | 0.000 | 0.000 | 0.397 | -- | -- | ***0.574*** | ***0.000*** | ***0.028*** | 0.000 | 0.000 | 0.000 |
| D15 | 4-QS | 0.001 | 0.000 | 0.001 | 0.000 | 0.000 | 0.001 | ***0.711*** | ***0.000*** | ***0.004*** | 0.222 | -- | -- | 0.060 | 0.000 | 0.001 | 0.000 | 0.000 | 0.000 |
| D22 | 4-QS | 0.000 | 0.000 | 0.000 | 0.000 | 0.000 | 0.000 | 0.000 | 0.000 | 0.000 | 0.252 | -- | -- | 0.023 | 0.000 | 0.006 | ***0.711*** | ***0.001*** | ***0.008*** |
| D29 | 4-QS | 0.000 | 0.000 | 0.000 | 0.000 | 0.000 | 0.001 | ***0.632*** | ***0.000*** | ***0.005*** | 0.300 | -- | -- | 0.060 | 0.000 | 0.000 | 0.000 | 0.000 | 0.001 |
| E1 | 5-XC | 0.003 | 0.000 | 0.000 | 0.000 | 0.000 | 0.000 | 0.000 | 0.000 | 0.000 | 0.029 | 0.000 | 0.000 | 0.001 | -- | -- | ***0.966*** | ***0.000*** | ***0.000*** |
| E2 | 5-XC | 0.002 | 0.000 | 0.000 | 0.000 | 0.000 | 0.000 | 0.000 | 0.000 | 0.000 | 0.023 | 0.000 | 0.000 | 0.263 | -- | -- | ***0.710*** | ***0.000*** | ***0.001*** |
| E5 | 5-XC | 0.000 | 0.000 | 0.000 | 0.047 | 0.000 | 0.000 | 0.051 | 0.000 | 0.000 | 0.000 | 0.000 | 0.000 | 0.343 | -- | -- | ***0.557*** | ***0.000*** | ***0.001*** |
| E6 | 5-XC | 0.000 | 0.000 | 0.000 | 0.000 | 0.000 | 0.000 | ***0.903*** | ***0.000*** | ***0.001*** | 0.000 | 0.000 | 0.000 | 0.028 | -- | -- | 0.067 | 0.000 | 0.000 |
| E7 | 5-XC | 0.000 | 0.000 | 0.000 | 0.000 | 0.000 | 0.000 | 0.000 | 0.000 | 0.000 | ***0.726*** | ***0.000*** | ***0.000*** | 0.273 | -- | -- | 0.000 | 0.000 | 0.000 |
| E10 | 5-XC | ***0.550*** | ***0.000*** | ***0.003*** | 0.052 | 0.000 | 0.001 | 0.000 | 0.000 | 0.000 | 0.295 | 0.000 | 0.000 | 0.097 | -- | -- | 0.001 | 0.000 | 0.000 |
| E13 | 5-XC | 0.000 | 0.000 | 0.000 | 0.000 | 0.000 | 0.000 | 0.001 | 0.000 | 0.000 | ***0.725*** | ***0.000*** | ***0.000*** | 0.274 | -- | -- | 0.000 | 0.000 | 0.000 |
| E17 | 5-XC | 0.001 | 0.000 | 0.000 | 0.003 | 0.000 | 0.000 | 0.000 | 0.000 | 0.000 | 0.302 | 0.000 | 0.000 | 0.043 | -- | -- | ***0.650*** | ***0.000*** | ***0.000*** |
| E19 | 5-XC | 0.010 | 0.000 | 0.000 | 0.000 | 0.000 | 0.000 | 0.000 | 0.000 | 0.000 | 0.362 | 0.000 | 0.000 | 0.000 | -- | -- | ***0.628*** | ***0.000*** | ***0.000*** |
| E20 | 5-XC | 0.033 | 0.000 | 0.000 | 0.000 | 0.000 | 0.000 | 0.030 | 0.000 | 0.000 | ***0.873*** | ***0.000*** | ***0.000*** | 0.061 | -- | -- | 0.004 | 0.000 | 0.000 |
| E21 | 5-XC | 0.000 | 0.000 | 0.000 | 0.000 | 0.000 | 0.000 | 0.004 | 0.000 | 0.000 | 0.025 | 0.000 | 0.000 | 0.283 | -- | -- | ***0.688*** | ***0.000*** | ***0.001*** |
| E23 | 5-XC | 0.000 | 0.000 | 0.000 | 0.000 | 0.000 | 0.000 | 0.003 | 0.000 | 0.000 | 0.229 | 0.000 | 0.001 | 0.204 | -- | -- | ***0.562*** | ***0.000*** | ***0.000*** |
| E26 | 5-XC | 0.343 | 0.000 | 0.000 | ***0.617*** | ***0.000*** | ***0.000*** | 0.029 | 0.000 | 0.000 | 0.002 | 0.000 | 0.000 | 0.000 | -- | -- | 0.009 | 0.000 | 0.000 |
| E27 | 5-XC | 0.000 | 0.000 | 0.000 | 0.000 | 0.000 | 0.000 | 0.000 | 0.000 | 0.000 | ***0.971*** | ***0.000*** | ***0.000*** | 0.014 | -- | -- | 0.015 | 0.000 | 0.000 |
| E28 | 5-XC | 0.138 | 0.000 | 0.000 | ***0.600*** | ***0.000*** | ***0.000*** | 0.161 | 0.000 | 0.000 | 0.000 | 0.000 | 0.000 | 0.000 | -- | -- | 0.101 | 0.000 | 0.000 |
| E29 | 5-XC | 0.000 | 0.000 | 0.000 | 0.096 | 0.000 | 0.001 | 0.000 | 0.000 | 0.000 | 0.019 | 0.000 | 0.000 | 0.308 | -- | -- | ***0.575*** | ***0.000*** | ***0.002*** |
| E30 | 5-XC | 0.000 | 0.000 | 0.000 | 0.000 | 0.000 | 0.000 | 0.021 | 0.000 | 0.000 | ***0.683*** | ***0.000*** | ***0.001*** | 0.194 | -- | -- | 0.099 | 0.000 | 0.001 |
| F1 | 6-DW | 0.000 | 0.000 | 0.000 | 0.000 | 0.000 | 0.000 | ***1.000*** | ***0.000*** | ***0.000*** | 0.000 | 0.000 | 0.000 | 0.000 | 0.000 | 0.000 | 0.000 | -- | -- |
| F10 | 6-DW | 0.352 | 0.000 | 0.000 | ***0.546*** | ***0.000*** | ***0.000*** | 0.000 | 0.000 | 0.000 | 0.099 | 0.000 | 0.000 | 0.000 | 0.000 | 0.000 | 0.003 | -- | -- |
| F16 | 6-DW | ***0.638*** | ***0.000*** | ***0.000*** | 0.000 | 0.000 | 0.000 | 0.362 | 0.000 | 0.000 | 0.000 | 0.000 | 0.000 | 0.000 | 0.000 | 0.000 | 0.000 | -- | -- |
| F19 | 6-DW | ***0.882*** | ***0.000*** | ***0.000*** | 0.118 | 0.000 | 0.000 | 0.000 | 0.000 | 0.000 | 0.000 | 0.000 | 0.000 | 0.000 | 0.000 | 0.000 | 0.000 | -- | -- |
| F21 | 6-DW | ***0.611*** | ***0.000*** | ***0.002*** | 0.103 | 0.000 | 0.001 | 0.008 | 0.000 | 0.001 | 0.003 | 0.000 | 0.000 | 0.000 | 0.000 | 0.001 | 0.271 | -- | -- |

Pr(0) means the individual was probably a migrant, Pr(1) means the parent was probably a migrant, Pr(2) means the grandparent was probably a migrant, and the combined immigration probability of pre-populations more than 0.50 were displayed by bold and itali.
